# The Prevalence of Esophageal Hypomotility and Its Relationship With Dysmotility Score in Vietnamese Patients Having Reflux Symptoms

**DOI:** 10.1002/jgh3.70330

**Published:** 2025-12-28

**Authors:** Hung Hoang Manh, Hue Luu Thi Minh, Trang Nguyen Thi Huyen, Phuong Do Nhat, Long Hoang Bao, Long Dao Van, Hang Dao Viet

**Affiliations:** ^1^ Hanoi Medical University Hanoi Vietnam; ^2^ Gastroenterology and Hepatology Center, Bach Mai Hospital Hanoi Vietnam; ^3^ The Institute of Gastroenterology and Hepatology Hanoi Vietnam; ^4^ College of Health Sciences, VinUniversity Hanoi Vietnam; ^5^ Endoscopy Centre, Hanoi Medical University Hospital Hanoi Vietnam

**Keywords:** esophageal hypomotility, FSSG, GERD, high‐resolution manometry

## Abstract

**Aim:**

This study aimed to determine the prevalence of esophageal hypomotility and examine its association with FSSG scores among Vietnamese patients presenting with reflux symptoms.

**Methods:**

A cross‐sectional study was conducted in 403 adults with reflux‐suspected symptoms and a GERD‐Q score ≥ 8 undergoing high‐resolution manometry (HRM) at Hoang Long Clinic (Hanoi, Vietnam). FSSG comprised two main components, including reflux score (FSSG‐R) and dysmotility score (FSSG‐M). Esophageal motility disorders were classified according to Chicago Classification v4.0; hypomotility disorders included ineffective esophageal motility (IEM) and absent contractility.

**Results:**

The mean FSSG total score and FSSG‐M score were 15.91 ± 5.94 and 8.03 ± 3.84, respectively. On manometry, 66.0% of patients had a diagnosis of esophageal hypomotility, predominantly IEM (63.5%). Esophagogastric junction (EGJ) hypotension and EGJ morphology type III were seen in 15.38% and 3.47%, respectively. There were no significant differences on FSSG scores between patients with normal esophageal motility and hypomotility. Linear regression analysis showed no correlation between the FSSG‐M score and distal contractile integral (DCI) or the number of ineffective swallows. In the multivariable logistic regression model, female and EGJ hypotension were significantly associated with the presence of esophageal hypomotility (aOR = 1.582 and 4.094, respectively).

**Conclusions:**

The prevalence of esophageal hypomotility among Vietnamese patients having reflux symptoms was high. FSSG total score and its dysmotility component score were not associated with the presence of esophageal hypomotility.

## 1 Introduction

Gastroesophageal reflux disease (GERD) is a prevalent gastrointestinal disorder, with a global prevalence of 12.92% and a rising trend, particularly in Asia [1]. In Southeast Asia, most GERD cases present as nonerosive reflux disease (NERD), which does not respond well with standard treatment on PPI as comparison with the erosive esophagitis group [2]. The pathogenesis of GERD is multifactorial, in which esophageal motility impairment plays an important role. A stepwise use of gastrointestinal functional and imaging testing such as ambulatory pH monitoring, endoscopy, and high‐resolution manometry (HRM) provides comprehensive characteristics of GERD; thus, it guides specific treatment. HRM is considered the gold standard for assessing sphincter pressure abnormalities and esophageal motility disorders (EMDs) [3]. The Lyon Consensus 2.0 highlights hypomotility disorders, including ineffective esophageal motility (IEM) and absent contractility, esophagogastric junction (EGJ) hypotension, and hiatal hernia as supportive evidence for GERD diagnosis [4]. In resource‐limited countries such as Vietnam, the use of HRM in evaluating esophageal motor function is restricted to several specialized centers. The lack of pathological evidence could hinder physicians optimizing and personalizing therapy for patients. In Vietnam, we have epidemiological data on motility disorders in patients with upper GI symptoms; however, studies using HRM to diagnose motility abnormalities specifically in GERD patients confirmed by 24‐h pH‐impedance monitoring have been limited by small sample sizes [5, 6, 7, 8].

Prokinetic agents are recommended in combination use for those who did not fully respond to acid suppression therapy, especially in patients with proven hypomotility disorders [9, 10]. The Frequency Scale for the Symptoms of GERD (FSSG) is a validated questionnaire comprising both acid reflux‐related and dysmotility‐related symptoms [11]. The FSSG has been validated in several Asian countries, including Japan and Indonesia, with studies demonstrating its correlation with reflux burden measured by endoscopy and its usefulness in screening for GERD in primary care settings [12, 13]. However, validation data comparing FSSG with HRM remain limited, particularly in Southeast Asia, where diagnostic resources for esophageal motility assessment are still scarce. A study over 163 Japanese patients having a diagnosis of GERD showed the FSSG score could predict the need for the addition of prokinetics to PPI therapy [14]. However, the relationship between FSSG scores and HRM‐defined hypomotility has not been well established. Moreover, no Vietnamese study has evaluated whether FSSG—an accessible tool in primary care—can serve as a surrogate indicator of esophageal hypomotility where HRM is limited. These findings raise the question of whether the FSSG score could be applied in primary care settings without HRM to indicate prokinetics for patients with GERD, particularly those with an incomplete response to initial PPI treatment. Therefore, this study aims *to evaluate the prevalence of esophageal hypomotility in patients having reflux symptoms and its relationship with FSSG score*.

## 2 Methods

### 2.1 Subjects

Inclusive criteria included patients older than 18 years old, had reflux‐suspected symptoms with a GERD‐Q (GERD questionnaire) score ≥ 8, and were indicated for HRM at Hoang Long Clinic and the Institute of Gastroenterology and Hepatology (Hanoi, Vietnam). Patients with a diagnosis of EGJ outflow disorders, distal esophageal spasm, or esophageal hypercontractile on HRM based on Chicago v4.0 were excluded. Patients with severe chronic conditions (heart failure, chronic kidney disease, cirrhosis) or patients with psychological disorders that prevent proper communication were also excluded from our study.

### 2.2 Data Collection

Patients who met the inclusion criteria provided consent before being interviewed, and data were collected using standardized forms. The study collected demographic characteristics, medical history including GERD and autoimmune systemic diseases (e.g., systemic lupus erythematosus, systemic sclerosis), clinical symptoms, clinical questionnaires including GERD‐Q, and FSSG, manometric and endoscopic findings. Participants were divided into two groups by their clinical symptoms: Group 1 included patients having esophageal symptoms (heartburn and regurgitation) according to the Montreal definition [15]. Group 2 included patients having both esophageal symptoms and extraesophageal symptoms (chest pain, sore throat, chronic cough, dyspnea, globus). Some overlapping atypical symptoms such as epigastric pain, belching, bloating, vomiting, nausea, dysphagia were reported in both groups. Body mass index (BMI) was also assessed and classified according to thresholds for Asian‐Pacific region [16].

### 2.3 GERD‐Q Score and FSSG Score

GERD‐Q and FSSG are two self‐administered questionnaires in approaching patients with reflux and reflux‐like symptoms and measuring response to treatment over time. GERD‐Q contains six questions with four questions containing positive predictors (scored from 0 to 3) and two questions containing negative predictors (reversely scored from 3 to 0). A cut‐off value of 8 points had a sensitivity of 71.4% and a specificity of 64.6% in diagnosing GERD [17]. FSSG consists of 12 questions, which were scored to indicate the frequency of symptoms as follows: never = 0, occasionally = 1, sometimes = 2, often = 3, and always = 4. FSSG comprised two main components including reflux score (FSSG‐R) and dysmotility score (FSSG‐M). When the cutoff score was set at 8 points, the FSSG showed a sensitivity of 62%, a specificity of 59%, and an accuracy of 60%, whereas a cutoff score of 10 points altered these values to 55%, 69%, and 63% [11].

### 2.4 EMDs

EMDs are diagnosed based on esophageal HRM using Chicago 4.0. HRM system used in our study was water‐perfused system (Laborie, Poland) with catheters containing 22 sensors. The cut‐off value of IRP was set at 19 mmHg by the manufacturer. The protocol was followed by Chicago 4.0, and all patients with suspected disorders were performed in an upright position [18]. All patients were performed multiple rapid swallows (MRS) to further evaluate suspected abnormalities. In cases where Achalasia Type I could not be ruled out, the Eckardt clinical score and esophageal barium swallow were performed to exclude this condition. EMDs are categorized as disorders of EGJ outflow or disorders of peristalsis. EGJ morphology was categorized into three types based on the distance between LES and crucial diaphragm (CD): type I (superimposed LES and CD), type II (a separation < 2 cm), and type III (a separation ≥ 2 cm) [19].

Lyon 2.0 consensus defined three supportive evidences for the diagnosis of GERD on HRM including esophageal hypomotility, EGJ hypotension and hiatal hernia [4]. Esophageal hypomotility comprises IEM and absent contractility presenting as the decrease in or loss of normal peristalsis in the context of normal LES relaxation. EGJ hypotension was defined when EGJ (or lower esophageal sphincter—LES) pressure less than 10mmHg [20]. Hiatal hernia on HRM was defined when having type III of EGJ morphology [21].

### 2.5 Data Analysis

The data were analyzed using Stata version 17.0. Descriptive statistics for qualitative data were presented as frequencies and percentages, while quantitative data were summarized as means (medians) and standard deviations (interquartile ranges). The Chi‐square test or Fisher's Exact Test was used to compare differences in proportions, and the One sample T‐test or Mann–Whitney test and Kruskal–Wallis test were used to compare differences in means. Linear regression and multivariate logistic regression were applied to identify factors associated with hypomotility findings. A *p* < 0.05 was considered statistically significant.

### 2.6 Research Ethical

The study was approved on ethical aspects under the code number IRB00003121 by the Institutional Review Board for Ethics in Biomedical Research of Hanoi Medical University (Vietnam). Participants were fully informed about the research procedures, and those who agreed to participate signed an informed consent form. Patients could withdraw at any time without facing any form of discrimination. The research findings were used solely for research purposes and were not used for any other purposes.

## 3 Results

A total of 415 patients met the selection criteria for inclusion in the study. After excluding 12 patients (05 with DES, 06 with EGJOO, and 01 with achalasia), 403 patients were included in the final analysis, including 242 females (60%). The mean age was 47.9 ± 11.9 (range: 19–75). A total of 49.4% of patients had a medical history of GERD, and endoscopic findings showed that 48.9% had reflux esophagitis. The mean total GERD‐Q score and FSSG score were 10.3 ± 1.9 and 15.9 ± 5.9, respectively. The two component scores of FSSG, including reflux score and dysmotility score, mean 7.9 ± 3.8 and 8.0 ± 3.8, respectively. A total of 93.3% of patients had an FSSG score ≥ 8 (Table 1).

**TABLE 1:** Patient characteristics (*n* = 403).

Variables	All patients (*n* = 403)	Group 1 (*n* = 351)	Group 2 (*n* = 52)	*p*
Sex (female), *n* (%)	242 (60.0)	211 (60.1)	31 (59.6)	0.95
Age, (mean, SD)	47.9 (11.9)	47.9 (11.9)	48.00 (11.89)	0.99
BMI (median, IQR)	21.9 (20.4–23.3)	21.8 (20.4–23.4)	21.9 (20.3–22.9)	0.60
BMI classification (*n*, %)				
Underweight (< 18.5)	25 (6.2)	20 (5.7)	5 (9.6)	0.57
Normal (18.5–22.9)	256 (63.5)	222 (63.3)	34 (65.4)	
Overweight (23–24.9)	85 (21.1)	75 (21.4)	10 (19.2)	
Obesity (≥ 25.0)	37 (9.2)	34 (9.7)	3 (5.8)	
Overlapping atypical symptom (*n*, %)				
Yes	353 (87.6)	306 (87.2)	47 (90.4)	0.51
No	50 (12.4)	45 (12.8)	5 (9.6)	
Medical history (*n*, *%*)				
GERD	199 (49.4)	165 (47.0)	34 (65.4)	0.013^b^
Systemic diseases^a^	3 (0.7)	2 (0.6)	1 (1.9)	0.29
Clinical questionnaires (median, IQR)				
Total GERD‐Q	10.00 (9.00–11.00)	10.00 (9.00–11.00)	11.0 (9.00–12.00)	0.017^c^
Total FSSG	16.00 (11.00–20.00)	16.00 (12.00–20.00)	13.00 (10.00–22.00)	0.179
FSSG‐R	8.00 (5.00–11.00)	7.00 (5.00–10.00)	8.00 (6.00–12.00)	0.197
FSSG‐M	8.00 (5.00–11.00)	8.00 (5.00–11.00)	6.00 (3.00–9.5)	0.007^c^
Total FSSG ≥ 8 (*n*, %)	376 (93.3)	326 (92.9)	50 (96.2)	0.38
Endoscopy findings (*n*, %)				
Barrett's esophagus	8 (1.9)	8 (2.3)	0 (0.0)	0.27
Hiatal hernia	9 (2.2)	7 (1.9)	2 (3.9)	0.39
Erosive esophagitis				
No	206 (51.1)	176 (50.1)	30 (57.7)	0.53
LA grade A	187 (46.4)	165 (47.0)	22 (42.3)	
LA grade B	9 (2.2)	9 (2.6)	0 (0.0)	
LA grade C‐D	1 (0.3)	1 (0.3)	(0.0)	
HRM findings				
Normal	137 (34.0)	113 (32.2)	24 (46.2)	0.14
IEM	256 (63.5)	229 (65.2)	27 (51.9)	
Absent contractility	10 (2.5)	9 (2.6)	1 (1.9)	
Resting UES pressure (mmHg) (Mean, SD)	55.1 (30.3)	55.3 (30.3)	53.8 (30.4)	0.65
Resting LES pressure (mmHg) (Mean, SD)	19.3 (10.3)	19.1 (10.2)	21.1 (11.1)	0.14
UES hypotension (< 33 mmHg) (*n*, %)	99 (24.6)	85 (24.2)	14 (26.9)	0.67
EGJ hypotension (< 10 mmHg) (*n*, %)	62 (15.4)	56 (19.9)	6 (11.5)	0.41
EGJ morphology (*n*, %)				
Type I	364 (90.3)	322 (91.7)	42 (80.8)	0.013^b^
Type II	25 (6.2)	17 (4.8)	8 (15.4)	
Type III	14 (3.5)	12 (3.4)	2 (3.9)	

Abbreviations: EGJ, esophagogastric junction; FSSG, frequency scale for symptoms of GERD; GERD‐Q, gastroesophageal reflux disease questionaire; IEM, ineffective esophageal motility; IQR, interquartile range; LA, Los Angeles classification; LES, lower esophageal sphincter; SD, standard deviation; UES, upper esophageal sphincter.

^a^ Systemic diseases included systemic lupus erythematosus and systemic sclerosis.

^b^ Chi‐square test.

^c^ Mann–Whitney test.

On HRM, the most common diagnosis was esophageal hypomotility disorders (66.0%), including 256 patients (63.5%) with IEM and 10 patients (2.5%) with absent contractility. UES hypotension and EGJ hypotension were presented in 24.6% and 15.4%, respectively. EGJ morphology was predominantly type I (90.3%), followed by type II (6.2%) and type III (3.5%) (Table 1).

**FIGURE 1:**
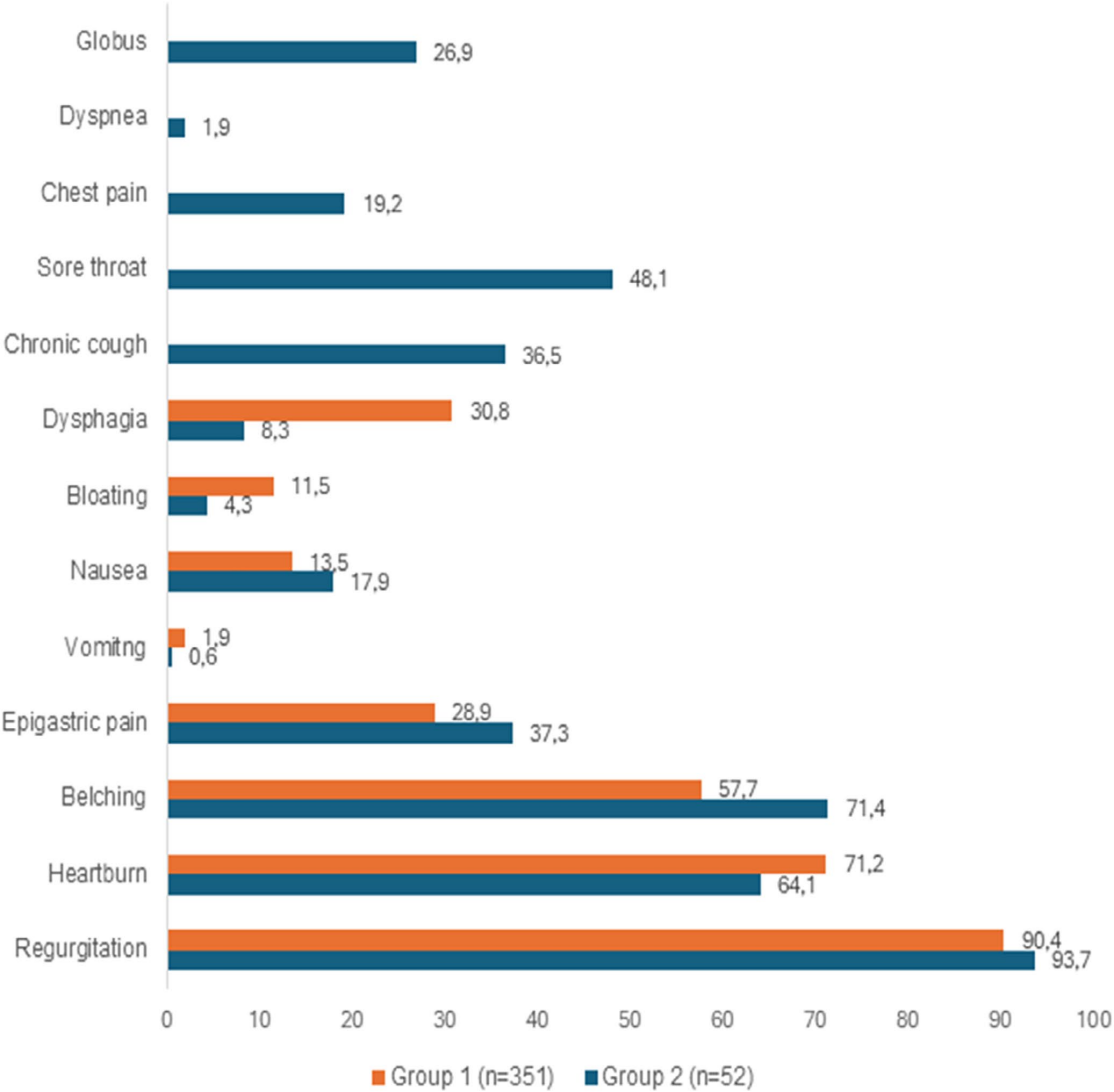
Clinical symptoms according to two different groups (*n* = 403).

The common clinical symptoms in patients were regurgitation (93.3%), belching (71.9%), heartburn (65.01%), and epigastric pain (36.2%) (Supporting Information). Among the participants, 12.9% presented with both esophageal and extra‐esophageal symptoms. Some atypical symptoms were also observed in both groups, which are presented in Figure 1. Patients in Group 1 and Group 2 showed overall similar HRM and endoscopy findings. There were no significant differences in sex or BMI classification among patient groups with different clinical symptoms. Patients in Group 2 had a higher prevalence of GERD history and EGJ morphology abnormalities compared to the other group (*p* = 0.013). The median GERD‐Q score of the patients in the study was 10 (9.00–11.00). The mean GERD‐Q scores in the patients of Group 2 were significantly higher compared to those in Group 1 (*p* = 0.017). In contrast, their mean FSSG‐M scores were lower than those of patients with only esophageal symptoms (*p* = 0.07). The median FSSG score of the participants was 16.00, with 93.3% of patients having an FSSG score of ≥ 8 points (Table 1).

**FIGURE 2:**
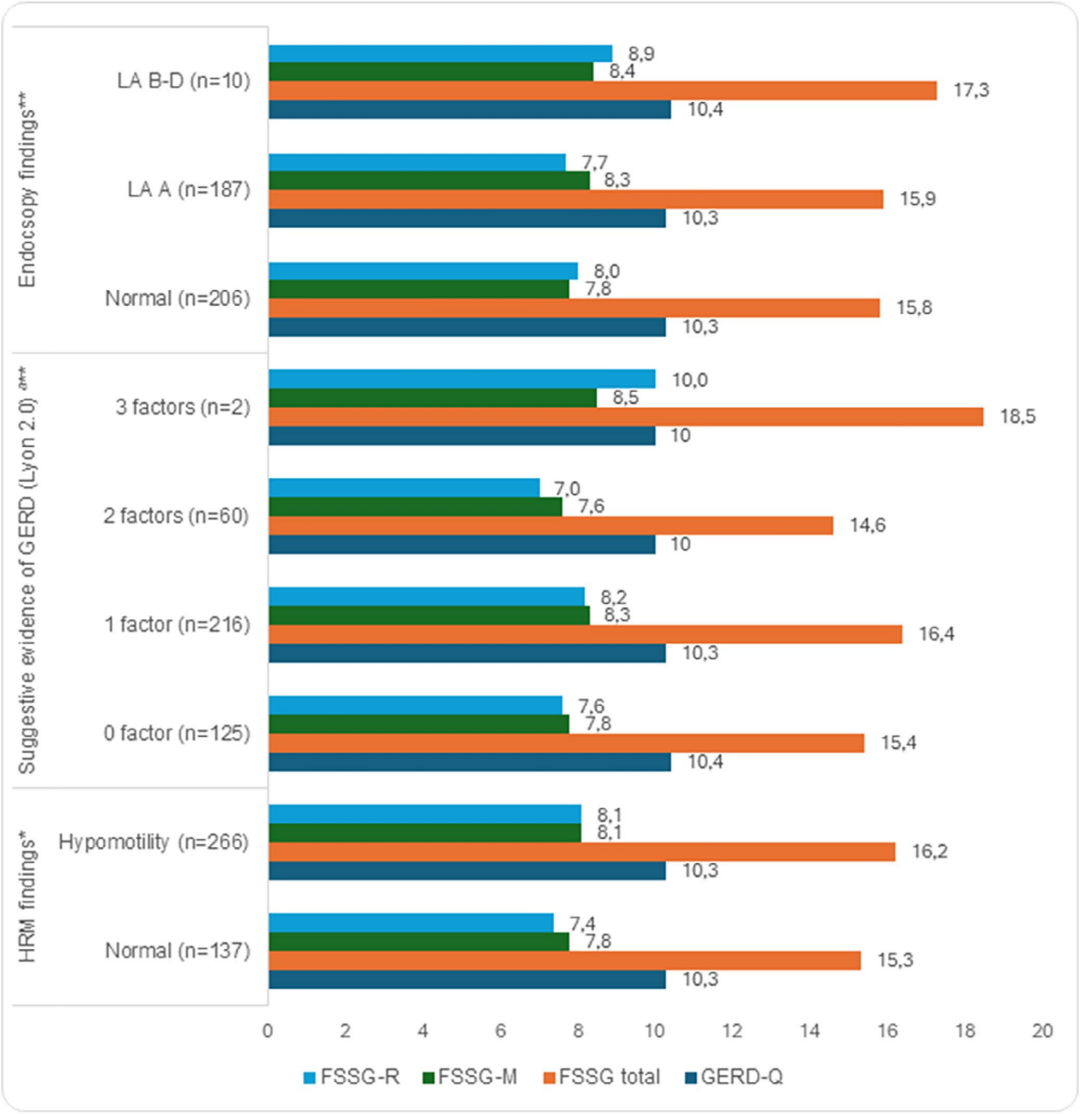
The relationship between clinical questionnaire scores and HRM findings, and Endoscopy findings (*n* = 403). ^ª^Suggestive evidence includes hypomotility, EGJ hypotension and hiatal hernia on HRM *Mann–Whitney test, **Kruskal–Wallis test, nonsignificant differences.

Comparing the clinical questionnaire scores between the normal motility group (*n* = 137) and the hypomotility group (*n* = 266), no statistically significant differences were observed between the two groups. There were no statistically significant differences in clinical scores between patients with endoscopic findings of reflux esophagitis and those evaluated according to the Lyon Consensus 2.0 criteria (Figure 2).

**FIGURE 3:**
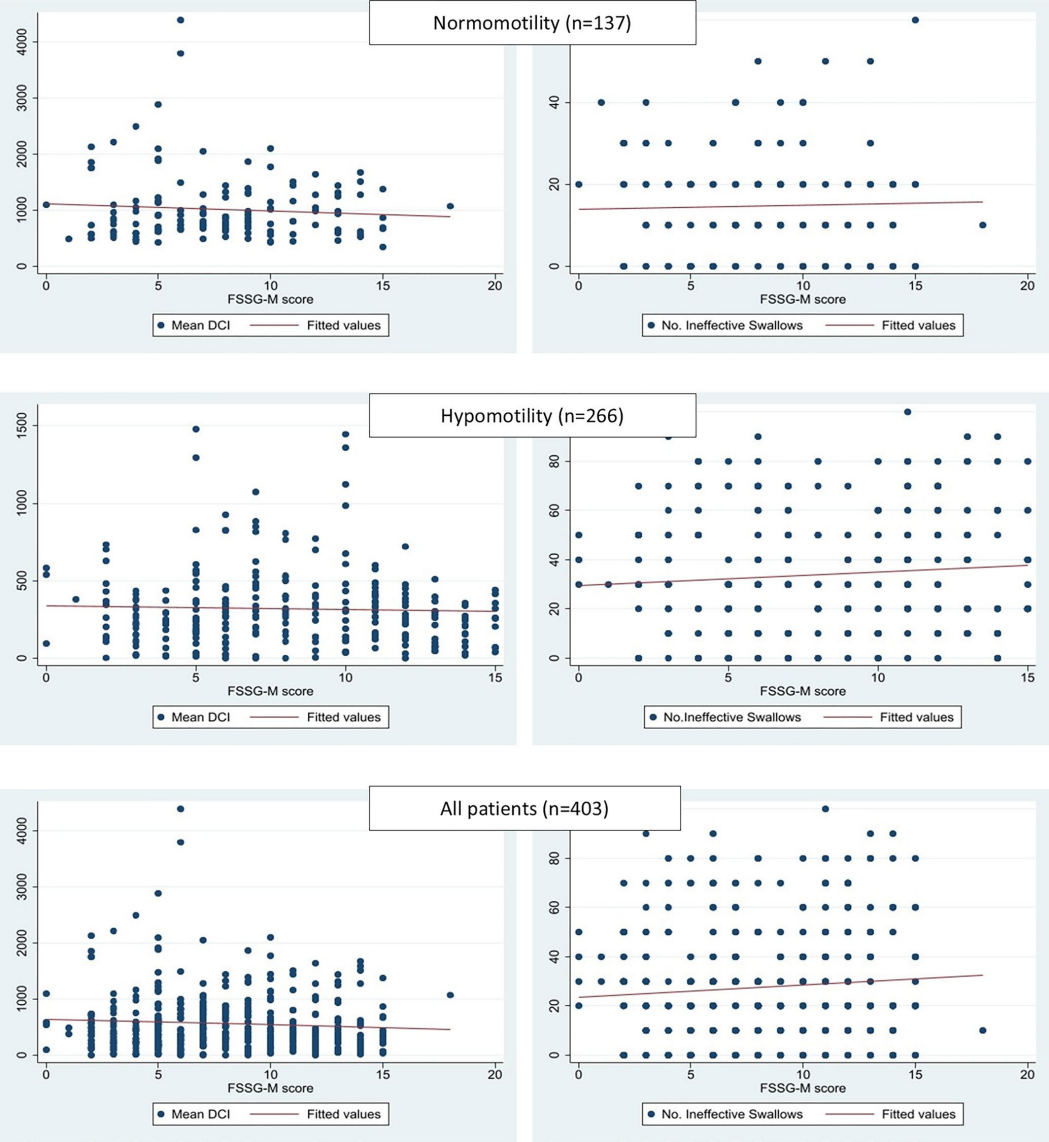
Correlation between clinical questionnaire scores and distal contractile integral and ineffective (*n* = 403).

Linear regression analysis showed no correlation between FSSG‐M score and distal contractile integral (DCI) or the number of ineffective swallows (Figure 3). Patients with EGJ hypotension exhibited a higher prevalence of esophageal hypomotility compared with those in the other groups (Table S1). In the multivariable logistic regression model (Table 2), female and LES hypotension and having esophageal symptoms were significantly associated with the presence of esophageal hypomotility (aOR = 1.69; 4.09 and 1.97, respectively).

**TABLE 2:** Multivariable logistic regression model of factors associated with hypomotility (*n* = 403).

Variables	aOR	*p*	95% CI
Female	**1.69**	***0.02***	***1.07–2.67***
Age	1.0	0.675	0.98–1.01
BMI classification			
Normal (18.5–22.9)	*Reference*	*Reference*	
Underweight (< 18.5)	0.89	0.788	0.35–2.18
Overweight (23–24.9)	0.70	0.21	0.40–1.22
Obesity (> 25.0)	0.70	0.36	0.33–1.50
History of GERD	1.45	0.10	0.93–2.28
Clinical symptom			
Group 2	*Reference*	*Reference*	
Group 1	***1.97***	***0.04***	***1.03–3.75***
GERD‐Q	1.00	0.97	0.88–1.14
FSSG	1.06	0.06	0.99–1.13
FSSG‐M	0.95	0.27	0.86–1.04
Reflux esophagitis			
Normal	*Reference*	*Reference*	
LA A	0.87	0.54	0.56–1.36
LA B‐D	5.10	0.14	0.59–43.80
EGJ hypotension	***4.11***	***< 0.01***	***1.92–8.82***
EGJ morphology			
Type I	*Reference*	*Reference*	
Type II	1.00	0.993	0.39–2.56
Type III	2.23	0.18	0.65–9.82

## 4 Discussion

Our study analyzed data from 403 patients to evaluate the relationship between FSSG score and esophageal motility parameters in patients with reflux symptoms.

### 4.1 Extraesophageal and Esophageal Hypomotility

Patients with abnormal esophageal motor function may impair the esophageal clearance function against refluxes, leading to reaching and irritating the laryngopharynx and causing troublesome symptoms [22, 23, 24]. Many studies also showed a high prevalence of esophageal hypomotility in patients presented with extraesophageal symptoms; however, the relationship between esophageal motility and reflux burden among this subject was inconsistent [23, 25]. Our study showed no significant differences in clinical scores, endoscopy, and manometric findings between patients with esophageal symptoms only and those with both esophageal and extraesophageal symptoms. All of the results could be explained by the perception of extraesophageal symptoms not only due to direct effects of mucosal alterations but also indirect effects of neurogenic, inflammatory, or behavioral pathways [26, 27], thus may vary between individuals regardless of the severity of reflux disease.

### 4.2 Manometry Findings and Symptom Severity

Previous studies agreed that esophageal hypomotility disorders are very common in patients with GERD [28, 29]. Similarly, our study presented more than half of patients had a diagnosis of esophageal hypomotility with the majority of IEM. Other suggestive evidence of GERD following Lyon 2.0 consensus including hypotensive EGJ and hiatal hernia (defined by EGJ morphology type III on HRM) were in lower cases (15.4% and 3.5%, respectively). The abnormalities in esophageal function and/or EGJ barrier have proven to be associated with a higher risk of erosive esophagitis [30]. However, the prevalence of esophagitis LA grades B–D in Vietnam and Asia‐Pacific region is reported very low, differently from other parts in the world [2, 5, 31]. Our study also reported that the combination of these suggestive factors did not increase the frequency and severity of symptoms presented by no differences on GERD‐Q and FSSG scores (Figure 2). These findings suggest that the severity of GERD symptoms is influenced not only by alterations in the antireflux barrier or mucosal damage, but also by other underlying mechanisms such as individual visceral hypersensitivity.

### 4.3 FSSG Score and Hypomotility

Our study showed no significant differences within HRM finding (normal vs. hypomotility disorders) and endoscopic finding (with or without reflux esophagitis) in terms of both FSSG‐M and FSSG total scores. FSSG‐M score was also not significantly correlated with DCI and the severity of hypomotility disorders defined by the number of ineffective swallows. Female and LES hypotension but not FSSG‐M or FSSG total score were associated with the presence of hypomotility disorders on HRM. A study over 394 patients with heartburn undergoing HRM in Japan also showed no difference on FSSG‐M between those having hypomotility disorders and those having normal motility [32]. In another study, FSSG‐M score was significantly lower for NERD than for reflux esophagitis in patients with FSSG total score ≥ 8 [33]. All these findings could imply that FSSG score and its components do not directly measure esophageal motor function but instead quantifies GERD‐related symptoms including both acid reflux and dysmotility.

Current guidelines do not clearly point out on which conditions prokinetic agents should be added on the therapy. In a narrative review published in 2021, the authors proposed an algorithm for decision making on prokinetic prescription in patients with esophageal hypomotility. Following this, prokinetics should be used when patients presented with dysphagia or reflux‐suspected symptoms and having manometric evidence of hypomotility disorders [9]. A study in Japan was conducted on 163 patients with GERD symptoms; it was the very first study performing to predict the need for prokinetics. The results suggested that combination therapy with prokinetics was effective for patients dissatisfied with PPI monotherapy and FSSG score is a useful predictor of the necessity for combination therapy [14]. Another study showed that the FSSG score could be used as a predictor of treatment response [34]. Therefore, FSSG score may not be used as a prediction of esophageal hypomotility disorders but is still a useful factor for considering adding prokinetic agents and predicting treatment outcome in patients with GERD. To date, FSSG cut‐off values have been developed and validated mainly for the diagnosis and follow‐up of GERD, with thresholds of 8–10 points showing modest sensitivity and specificity for endoscopy‐proven GERD [11]. Our findings suggest that FSSG should be interpreted as a tool to quantify overall GERD‐related symptom burden. Even in the absence of a direct correlation with HRM metrics, higher FSSG scores may still help identify patients who are more likely to benefit from step‐up therapy, including the addition of prokinetics, especially in primary care settings where HRM is not readily available. Future studies integrating HRM, 24‐h pH monitoring, and FSSG score may elucidate their association with clinical clusters and improve treatment decision‐making, especially in resource‐constrained settings. Given the limited evidence on the relationship between FSSG scores and esophageal hypomotility and the known geographic variation in GERD epidemiology, we also propose that future research aim to establish region‐specific FSSG cut‐off values relevant to esophageal hypomotility.

On the other hand, patients in the hypomotility group in this study reported a high prevalence of overlapping symptoms such as nausea, vomiting, bloating, and epigastric pain (Figure S1). These symptoms are suggestive of underlying disorders of gut–brain interaction (DGBIs). The coexistence of DGBIs—particularly functional heartburn, reflux hypersensitivity, and functional dyspepsia—can substantially influence the interpretation of GERD‐related symptoms and diagnostic metrics [35, 36]. A recent systematic review and meta‐analysis reported that the prevalence of GERD–FD overlap is approximately 7.41% (95% CI: 4.55%–11.84%) in the general population [37]. A study from Vietnam among 295 patients presenting with reflux symptoms reported that 46.8% had overlapping functional dyspepsia symptoms [38]. In GERD patients, reflux pathogenesis such as impaired acid clearance, transient LES relaxations, LES hypotension, and IEM often contribute directly to symptom generation [39]. Therefore, a correlation between clinical symptoms (as assessed by the FSSG score) and hypomotility is expected in this group. In contrast, patients with functional heartburn or reflux hypersensitivity frequently demonstrate normal acid exposure and preserved motility despite reporting significant symptoms. For these individuals, visceral hypersensitivity, altered central pain processing, and psychosocial factors might play a more dominant role than dysfunction of the esophagus [40]. Taken together, DGBI overlap complicates the clinical interpretation of manometry findings by decoupling symptoms from physiology and reducing the specificity of FSSG for predicting GERD.

### 4.4 Study Limitations

This study was a single‐center, cross‐sectional descriptive study without 24‐h pH monitoring so that pathologic GERD diagnosis based on Lyon 2.0 criteria could not be confirmed, and symptom presentation may have overlapped with other esophageal or functional disorders. The study also did not collect information on the duration of other symptoms such as belching and nausea; therefore, it did not meet the criteria required to diagnose other DGBIs. Future studies should conduct a combined approach of HRM measurements, pH impedance measurements, and detailed assessment of clinical symptoms duration to further explore the associations. Evidence from this approach would help clarify whether distinct motility patterns or symptom clusters can reliably differentiate GERD from functional heartburn, reflux hypersensitivity, and provide more appropriate diagnostic and treatment strategies. Another concern is that our study utilized a water‐perfusion HRM system, which might affect the classification of EMDs. To mitigate this limitation, we strictly followed the Chicago 4.0 protocol. Further research may be performed on solid catheter systems to more accurately classify the disorders.

## 5 Conclusion

The prevalence of esophageal hypomotility among Vietnamese patients having reflux symptoms was high. FSSG total score and its dysmotility component score were not associated with the presence of esophageal hypomotility.

## Funding

The authors have nothing to report.

## Conflicts of Interest

The authors declare no conflicts of interest.

## Supporting information


**Figure S1:** Patient's clinical symtoms (*n* = 403).
**Table S1:** HRM findings in participants.

## Data Availability

The data that support the findings of this study are available from the corresponding author upon reasonable request.
