# Supplementary material for: The Prevalence of Esophageal Hypomotility and Its Relationship With Dysmotility Score in Vietnamese Patients Having Reflux Symptoms
Source: JGH Open. 2025 Dec 28;9(12):e70330. doi: 10.1002/jgh3.70330 (PMC12745500; doi:10.1002/jgh3.70330)
Supplement: Supplementary file 1 — Figure S1: Patient's clinical symtoms (n = 403). Table S1: HRM findings in participants. [file JGH3-9-e70330-s001.docx]

# Supplementary

Figure S1. Patient‘s clinical symtoms (n=403)

Table S1. HRM findings in participants

| **Characteristics** | **HRM findings (n=403)** | | **p** |
| --- | --- | --- | --- |
|  | **Hypomotility** | **Normal** |  |
| *Resting UES pressure (mmHg) (Mean, SD)* | 54.9 (28.37) | 55.43 (33.76) | 0.42 |
| *Resting LES pressure (mmHg) (Mean, SD)* | **17.4 (9.26)** | **23.1 (11.24)** | **<0.01** |
| *UES hypotension (<33mmHg) (n,%)* | 62 (62.6) | 37 (37.4) | 0.41 |
| *EGJ hypotension (<10 mmHg) (n,%)* | **53 (86.5)** | **9 (14.5)** | **<0.01** |
| **EGJ morphology (n,%)** | | | |
| *Type I* | 238 (65.4) | 126 (34.6) | 0.579 |
| *Type II* | 17 (68.0) | 8 (32.0) |  |
| *Type III* | 11 (78.6) | 3 (21.4) |  |
